# Bridging ethical and governance gaps in digital health: insights from users and startups in Iran

**DOI:** 10.1186/s12913-026-14439-9

**Published:** 2026-04-25

**Authors:** Nafiseh Salehnia

**Affiliations:** Department of Economic Development and Planning, Faculty of Management and Economics, Tarbiat Modares University, Tehran, Iran

**Keywords:** Digital health governance, Ethical accountability, Data governance, Iran, Rregulatory fragmentation, Health policy, Sustainability

## Abstract

**Background:**

Digital health adoption is accelerating in many low- and middle-income countries, yet governance, ethical safeguards, and data stewardship often lag behind technological uptake. This mismatch can undermine user trust, service quality, and the long-term sustainability of digital health innovations. This study aimed to identify and conceptualize key governance gaps influencing the development of Iran’s digital health ecosystem.

**Methods:**

A qualitative thematic analysis was conducted using 18 semi-structured interviews with policymakers, entrepreneurs, and healthcare professionals, alongside content analysis of 700 user comments extracted from major Iranian digital health platforms. Data were analyzed through the six-phase framework of Braun and Clarke, combining inductive (data-driven) and deductive (theory-informed) coding. Triangulation between interview and user datasets enhanced validity, and the COREQ checklist guided reporting.

**Results:**

Six interrelated governance gaps were identified: (1) Regulatory and Oversight Gap – fragmented authorities and lack of unified licensing; (2) Ethical and Trust Gap – weak accountability and declining user confidence; (3) Data Governance Gap – unclear data ownership and poor privacy protection; (4) Quality and Standardization Gap – lack of evaluation indicators and accreditation systems; (5) Institutional Capacity Gap – limited expertise and low readiness for digital transformation; and (6) Economic and Sustainability Gap – insufficient financial incentives and policy support. Findings from user comments confirmed that governance weaknesses directly affect satisfaction, perceived fairness, and trust in digital health services.

**Conclusions:**

Digital health in emerging settings requires a multi-level governance approach that aligns national policy, institutional regulation, and operational accountability. We propose an integrated macro–meso–micro governance model with cross-cutting principles (transparency, responsibility and data integrity, participation, trust) to guide policymakers and platform operators. Strengthening a unified regulator, a national ethics charter for digital health, and a health data governance framework are priority actions.

**Supplementary Information:**

The online version contains supplementary material available at 10.1186/s12913-026-14439-9.

## Background

The diffusion of digital technologies—including telemedicine, mobile health (mHealth) applications, electronic health records (EHRs), and selected AI-supported tools—has reshaped health service delivery in many settings, with the potential to improve access, efficiency, and patient engagement [1]. At the same time, the expansion of digital health introduces complex governance, ethical, and data stewardship challenges. If inadequately addressed, these challenges may undermine public trust, compromise patient safety, and exacerbate existing inequalities [2].

International organisations and policy frameworks increasingly emphasise that the effectiveness and sustainability of digital health systems depend not only on technological capability but also on robust governance arrangements. Guidance from the World Health Organization and the OECD highlights the importance of coherent national frameworks for health data governance, clearly defined accountability mechanisms, and the institutionalisation of ethical principles within digital health policy and regulation [3–4]. In the absence of such arrangements, countries may experience fragmented oversight, opaque data practices, and declining user confidence.

Ethical considerations have long been central to health system governance. Classical bioethical principles—autonomy, beneficence, non-maleficence, and justice—remain highly relevant in digital contexts where clinical responsibility, algorithmic mediation, and large-scale data use intersect [5]. In parallel, perspectives from information ethics and data governance underscore the role of data integrity, transparency, and accountability as foundational elements of trustworthy digital health systems [6].

Governance challenges tend to be particularly pronounced in low- and middle-income countries (LMICs), where technological innovation may advance more rapidly than institutional adaptation and regulatory enforcement capacity [7]. In such contexts, fragmented oversight structures, resource constraints, and limited ethical guidance can create systemic governance gaps that weaken both the legitimacy and sustainability of digital health initiatives.

A growing body of international research has examined digital health governance, focusing on how ethical, organisational, and regulatory factors shape the equitable delivery of technology-enabled care. Studies published in BMC Health Services Research and related journals have highlighted barriers such as digital exclusion among vulnerable populations, variability in institutional readiness, and the importance of transparent data governance and accountability for adoption and safety [8–10]. Research on telemedicine implementation, for example, has documented how organisational capacity, leadership, and regulatory clarity influence service quality and user acceptance [9–11]. Together, these strands of literature situate governance deficits as a central determinant of both practical performance and ethical integrity in digital health systems.

In Iran, digital health services have expanded unevenly over recent years, particularly in the form of telemedicine platforms and health-related applications. This expansion has occurred within a broader context characterised by economic constraints, regulatory complexity, and evolving institutional arrangements. Available national evidence points to persistent governance and ethical challenges. Qualitative research on e-health businesses has documented legal and organisational barriers, including fragmented regulation, non-standardised licensing processes, and weak monitoring mechanisms, which constrain compliance and sustainable operation [12]. Complementary studies focusing on user experiences have reported concerns related to service quality, platform reliability, lack of accountability (such as payment without service or refund), and privacy protection [13]. These findings suggest that governance and ethical gaps affect both providers and users within Iran’s digital health ecosystem.

Although shaped by specific institutional and political conditions, many of these challenges reflect patterns observed across LMICs more broadly, including overlapping regulatory mandates, unclear data ownership, limited grievance mechanisms, and weak coordination across governance levels [7, 14]. These observations motivate the present study’s focus on examining governance and ethical barriers through a multi-stakeholder lens and on developing an integrated governance framework suited to an emerging economy context. The framework is designed to conceptually align macro (policy), meso (regulatory), and micro (operational) levels, while foregrounding cross-cutting ethical principles—transparency, responsibility, data integrity, stakeholder participation, and trust—frequently highlighted in global governance guidance [2, 4].

Despite extensive international discussion of ethical and governance challenges in digital health, evidence from LMIC settings remains fragmented and often limited to single stakeholder groups or narrow technological dimensions. In Iran, existing studies have tended to focus separately on regulatory issues, technical implementation, or descriptive accounts of user experiences. This study addresses this gap by (i) triangulating interviews with startups and policy experts with user-generated comments to capture both supply- and demand-side perspectives, (ii) providing context-specific qualitative evidence on how governance gaps are experienced and operationalised in practice, and (iii) synthesising these insights into a policy-oriented, multi-level governance framework.

The aim of this study was to explore and synthesise perceived ethical and governance challenges in Iran’s digital health ecosystem from the perspectives of users, startups, and policy experts, and to develop an analytically derived, policy-oriented multi-level framework informed by the qualitative findings.

In this study, “digital health” refers primarily to telemedicine and online consultation platforms, health information and service applications, and platform-based health service marketplaces used by the public and startups in Iran. The analysis does not encompass wearable or implantable devices, hospital information systems and EHR implementation, or clinical AI decision-support systems, as these domains were outside the scope of the selected platforms and the participant sampling frame.

## Methods

### Study design

This research employed a qualitative exploratory design to investigate the governance and ethical challenges experienced by both users and firms operating in Iran’s digital health sector. A qualitative approach was appropriate given the complexity and contextual nature of governance and ethical phenomena, which require understanding participants’ experiences and perceptions in depth rather than measurement or hypothesis testing [15].

The study was conducted between September 2021 and May 2024 and followed the *Consolidated Criteria for Reporting Qualitative Research (COREQ)* guidelines to ensure transparency and rigor [16].

### Setting and context

Iran’s digital health ecosystem has rapidly evolved, with numerous startups offering online medical consultations, telepharmacy services, and health information systems. However, as previous studies have shown, this growth has occurred within a fragmented policy and regulatory environment, characterized by overlapping mandates and the absence of a unified digital health regulator. The study setting was chosen to reflect these systemic complexities and to capture multiple stakeholder perspectives within the ecosystem.

### Participants and sampling

Two groups of participants were targeted to capture diverse viewpoints:

1. **Digital health entrepreneurs and managers** (*n* = 13) from startups or small firms providing telemedicine or health information services;
2. **Policy experts and healthcare professionals** (*n* = 5), including officials, academic specialists, and digital governance consultants.

We used purposive sampling to recruit (a) digital health startup founders/managers and (b) policy experts/practitioners with experience in digital health governance. Inclusion criteria were: (i) at least 2 years of relevant professional experience, (ii) direct involvement in digital health platform development/regulation/policy implementation, and (iii) willingness to provide informed consent. We sought diversity in startup business models (e.g., B2C/B2B/public-sector contractors) and policy roles (e.g., Ministry of Health/regulatory bodies/health IT governance). Recruitment was stopped when no substantively new codes emerged in the final interviews and the research team judged thematic saturation to have been achieved.

An overview of participant characteristics is provided in Table 1.

**Table 1:** Participant characteristics (interviews, *n* = 18)

ID	Stakeholder group	Role	Organisation / Platform (type)	Domain	Years of experience	Location
P1	Startup	Founder / Manager	telemedicine platform	Telemedicine	3–5	Tehran
P2	Startup	Founder / Manager	Telemedicine platform	Telemedicine	3–5	Tehran
P3	Startup	Founder / Manager	Women’s health app	mHealth application	3–5	Tehran
P4	Startup	Founder / Manager	Digital health platform	Digital health services	3–5	Yazd
P5	Startup	Founder / Manager	Health service platform	Digital health services	3–5	Tehran
P6	Startup	Founder / Manager	Health information platform	Health information services	3–5	Tehran
P7	Startup	Founder / Manager	Health IT solutions	Health IT	3–5	Tehran
P8	Startup	Board member / Expert	Iranian Telemedicine Association	Telemedicine governance	3–5	Tehran
P9	Startup	Founder / Manager	Telemedicine platform	Telemedicine	3–5	Mashhad
P10	Startup	Founder / Manager	Health service platform	Digital health services	2–5	Tehran
P11	Startup	Founder / Manager	Telemedicine platform	Telemedicine	5–10	Tehran
P12	Startup	Founder / Manager	Health service platform	Digital health services	2–5	Kerman
P13	Startup	Founder / Manager	Digital health media/services	Digital health services	5–10	Tehran
P14	Healthcare professional	Senior official	Ministry of Health	Health policy / regulation	10–15	Tehran
P15	Policy expert	Regulatory expert	Psychology & Counselling Organization	Professional regulation	5–10	Tehran
P16	Policy expert	Senior official	Ministry of Health	Digital health governance	10–15	Tehran
P17	Policy expert	Policy analyst	National Center for Cyberspace	Digital governance	5–10	Tehran
P18	Policy expert	Board member / expert	Iran Computer Guild Organization	ICT & platform regulation	10–15	Tehran

Participants were primarily based in Tehran, reflecting the concentration of digital health governance and platform headquarters in the capital; however, the inclusion of startups from Yazd, Mashhad, and Kerman enhanced geographic diversity and transferability of findings

### Data collection

Data collection involved two complementary sources:

- **Semi-structured interviews** (*n* = 18):

The interviews were originally conducted as part of a national policy project commissioned by the National Center for Cyberspace, focusing on ethical, regulatory, and operational challenges in digital health businesses. The interview guide—now provided as Additional File 1—included 29 semi-structured questions covering:

- User–platform interactions and dispute resolution;
- Licensing and oversight mechanisms;
- Data governance, privacy, and informed consent;
- Service quality, provider verification, and platform accountability;
- Institutional capacity, security, and pricing policies;
- Ethical guidelines and implementation barriers;
- Additional open-ended questions on organizational challenges.

Interviews lasted 45–60 min, were conducted in Persian (face-to-face or via secure online platforms), audio-recorded with verbal consent, and transcribed verbatim.

- **User-generated comments: data sources and selection**

We analysed approximately 700 publicly available user comments related to digital health applications and platforms. Comments were collected from **CafeBazaar**,** Google Play**,** and Myket**, which are the three most widely used mobile application marketplaces in Iran, between **2021 and 2024**. Applications were selected based on their visibility and user engagement on these platforms (e.g., number of downloads and user reviews), with a focus on telemedicine, online consultation, and health service applications.

**Inclusion criteria** were: (i) comments written in Persian, (ii) substantive content reflecting user experiences or concerns related to service quality, privacy, data use, accountability, safety, or platform governance, and (iii) posting within the specified timeframe.

**Exclusion criteria** included duplicate entries, spam or promotional content, one-word or non-informative comments, and content unrelated to digital health service use. The unit of analysis was the individual user comment.

#### Ethical considerations for user comments

All comments were publicly accessible at the time of data collection. Usernames and any potentially identifying information were removed prior to analysis. When illustrative examples are presented, quotations are kept brief and anonymised to minimise any risk of identification.

An overview of the user-generated comment dataset and its key characteristics is presented in Table 2.

**Table 2:** Overview of user-generated comment dataset

Characteristic	Description
Platforms	CafeBazaar, Google Play, Myket
Time period	2021–2024
Types of applications	Telemedicine, online consultation, digital health service platforms
Total comments analysed	≈ 700
Unit of analysis	Individual user comment
Language	Persian
Exclusion criteria	Duplicates, spam/promotional content, non-informative or irrelevant comments
Ethical handling	Public data; usernames removed; anonymised excerpts

### Data analysis

The data were analyzed using the six-phase thematic analysis framework proposed by Braun and Clarke [17]. Both inductive (data-driven) and deductive (theory-informed) coding strategies were applied to identify governance and ethical barriers in digital health.

**Fig. 1:**
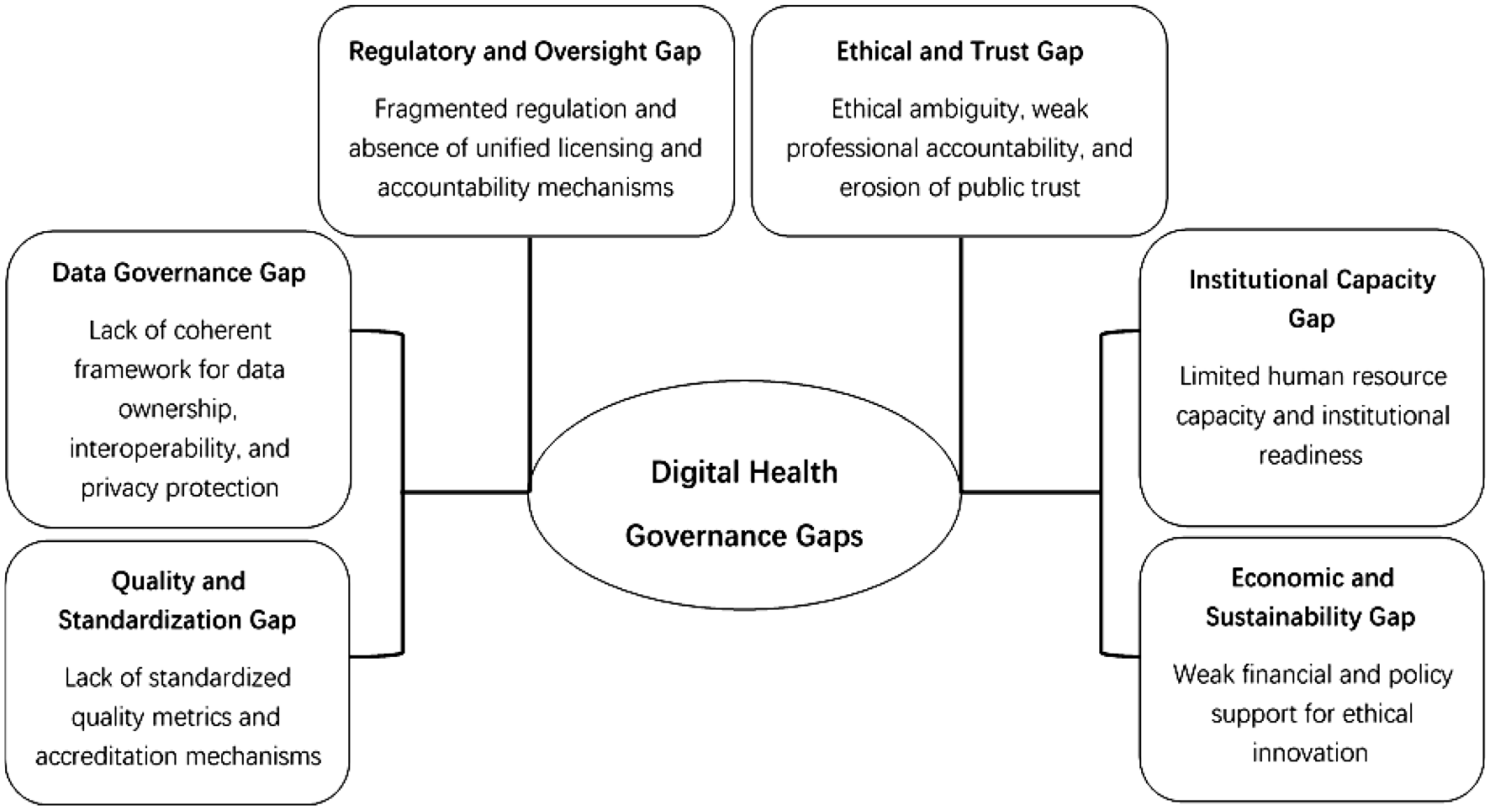
Digital health governance gaps identified through thematic analysis of interviews and user-generated comments. The figure summarises six major governance gaps shaping Iran’s digital health ecosystem, spanning regulatory, ethical, institutional, data-related, quality, and economic domains. The gaps are analytically derived from qualitative findings and illustrate areas of systemic weakness in oversight, accountability, and service delivery

1. **Familiarization**: All interview transcripts and textual data were read repeatedly to gain a comprehensive understanding of participants’ perspectives.
2. **Generating initial codes**: Relevant segments of text were systematically coded to capture issues related to governance, ethics, regulation, and data practices.
3. **Searching for themes**: Codes were organized into broader categories reflecting emerging patterns in users’ and providers’ experiences.
4. **Reviewing themes**: Preliminary themes were refined through iterative comparison across data sources to ensure internal consistency and distinctiveness.
5. **Defining and naming themes**: Each theme was clearly defined and labeled, resulting in six overarching governance domains that captured systemic weaknesses.
6. **Producing the report**: Final themes were synthesized and visualized in a conceptual map (Fig. 1), linking governance gaps to the integrated multi-level framework proposed in this study.

To enhance rigor and credibility, triangulation was achieved by analyzing interview and user data in parallel, and patterns were cross-validated between the two sources [15]. Reflexive memos were maintained throughout to document analytical decisions and ensure transparency [15, 17].

### Trustworthiness and reflexivity

To enhance the credibility and rigor of the study, we followed Lincoln and Guba’s (1985) four criteria for qualitative quality.

- **Credibility** was strengthened through triangulation of data sources and limited member checking with three participants.
- **Transferability** was supported by providing rich descriptions of the research context and participant characteristics.
- **Dependability** was enhanced through maintaining an audit trail of coding decisions and peer debriefing with external qualitative researchers.
- **Confirmability** was supported through reflexive journaling and independent review of coding decisions.

The researcher adopted a reflexive stance, acknowledging her prior experience in health governance research while remaining attentive to emergent themes and alternative interpretations.

### Coding process and peer review

Data analysis was conducted using **MAXQDA 2020**. All interview transcripts and user-generated comments were coded primarily by the first author through an iterative process of open coding, codebook development, and thematic refinement.

To enhance analytic rigor, two external qualitative researchers with expertise in health policy and qualitative methods conducted peer debriefing and an audit of a subset of the coded data. This review focused on code coherence, theme boundaries, and alternative interpretations. Feedback from this process was incorporated into revisions of the codebook and theme structure. Disagreements were resolved through discussion until analytic consensus was reached.

### Triangulation and integration of data sources

A convergent qualitative design was used to integrate interview data and user-generated comments. Interview transcripts and user comments were analysed using a shared thematic framework. Initial codes were developed inductively from interview data and iteratively refined through coding of user comments.

Triangulation was operationalised by examining whether user comments (i) confirmed, (ii) nuanced, or (iii) challenged themes identified in interviews. Divergent patterns were discussed and used to refine theme definitions and boundaries. The final themes reflect convergent and complementary insights across both data sources, while preserving source-specific perspectives where relevant.

## Results

Thematic analysis of interview data and user-generated comments identified six major governance gaps shaping the development and operation of Iran’s digital health ecosystem (Fig. 1). These gaps span regulatory, ethical, institutional, and operational domains and were consistently reflected across both stakeholder interviews and user experiences.

### Regulatory and oversight gap

Participants consistently reported fragmented regulatory structures and overlapping jurisdictions between the Ministry of Health, the Ministry of ICT, and other supervisory bodies. Startup representatives described uncertainty regarding licensing responsibilities and inconsistent enforcement practices across institutions.

One startup founder explained:

Each institution has its own set of rules. You get one license and still need another from a different agency. In some cases, no one can clearly tell you which authority is responsible. (Startup interview)

Another participant noted:

We were told there is no clear regulation in advertising, but after we started operating, we faced disciplinary actions. The rules are unclear and interpreted differently by each organisation. (Startup interview)

User comments indirectly reflected these regulatory ambiguities, particularly in relation to accountability and service validity:

I booked an appointment through the app, but the clinic said online appointments were not valid. If there is no contract with the doctor, why do you waste people’s time? (User comment)

### Ethical and trust gap

The absence of clear and enforceable ethical guidelines for online medical services emerged as a major concern. Users frequently raised issues related to privacy, misleading information, and perceived conflicts of interest in online consultations.

Several users expressed doubts about trustworthiness:

I don’t trust these platforms anymore. You don’t know who sees your information, and there is no way to be sure how doctors are monitored. (User comment)

Another user stated:

Only positive comments are shown. If you complain, your comment never appears. (User comment)

Startup participants also acknowledged that the lack of formal ethical standards complicates accountability:

There is no unified ethical framework for online services. Each platform decides for itself, and that creates confusion for both doctors and users. (Startup interview)

### Data governance gap

Uncertainty surrounding data ownership, storage, and access was identified as a critical governance gap. Participants highlighted the absence of clear rules on where health data should be stored and who retains control over patient records.

One startup manager stated:

Health data should not belong to applications. In our view, data should be stored centrally and governed by the state, not by individual platforms. (Startup interview)

Concerns regarding data security and foreign data storage were also raised:

Some platforms use foreign servers because there is no secure domestic infrastructure. This creates serious risks for privacy and data protection. (Startup interview)

Users echoed these concerns, particularly regarding control over personal information:

There is no option to delete past consultations. This violates privacy because anyone who accesses your phone can see your medical history. (User comment)

### Quality and standardization gap

Participants and users consistently reported the absence of standardized benchmarks for online consultations and service delivery. Users described delayed responses, premature termination of consultations, and lack of follow-up mechanisms.

One user commented:

You send a message in the morning and sometimes get a reply late at night. The doctor seems rushed and ends the consultation quickly, even though the consultation fee is not low. (User comment)

Another user noted:

After the doctor prescribed medication, the conversation was closed. If the medicine causes side effects, there is no way to follow up. (User comment)

Startup participants acknowledged the lack of formal standards:

There is no regulation defining how long an online consultation should last or whether prescribing medication in text-based consultations is appropriate. (Startup interview)

### Institutional capacity gap

Limited institutional capacity in areas such as digital ethics, health informatics, and data protection was a recurring theme. Policymakers and startup representatives described shortages of specialised expertise and weak coordination mechanisms across organisations.

One policy expert stated:

There is a serious lack of trained personnel who understand both health and digital governance. Most institutions are still learning through trial and error.(Policy expert interview)

Another participant highlighted organisational resistance:

Even when guidelines exist, there is resistance to change. Departments work in silos and coordination is very weak. (Startup interview)

### Economic and sustainability gap

Economic constraints and unclear reimbursement mechanisms were identified as barriers to ethical and sustainable operation. Entrepreneurs described tensions between ethical compliance and financial viability.

One startup founder explained:

Investing in secure systems or ethical review processes slows us down. There is no financial incentive for doing the right thing. (Startup interview)

Users frequently complained about pricing, lack of insurance coverage, and refund policies:

I paid for the consultation, but no service was provided. The money was deducted, and no one responded. (User comment)

### Summary of governance gaps

Taken together, these six governance gaps illustrate systemic weaknesses in the regulation and oversight of digital health services. Evidence from both stakeholder interviews and user-generated comments indicates that regulatory fragmentation, weak ethical guidance, and limited institutional capacity interact to undermine service quality, trust, and sustainability. Figure 1 summarises these interrelated gaps.

### Integrating the governance gaps: a multi-level framework

**Fig. 2:**
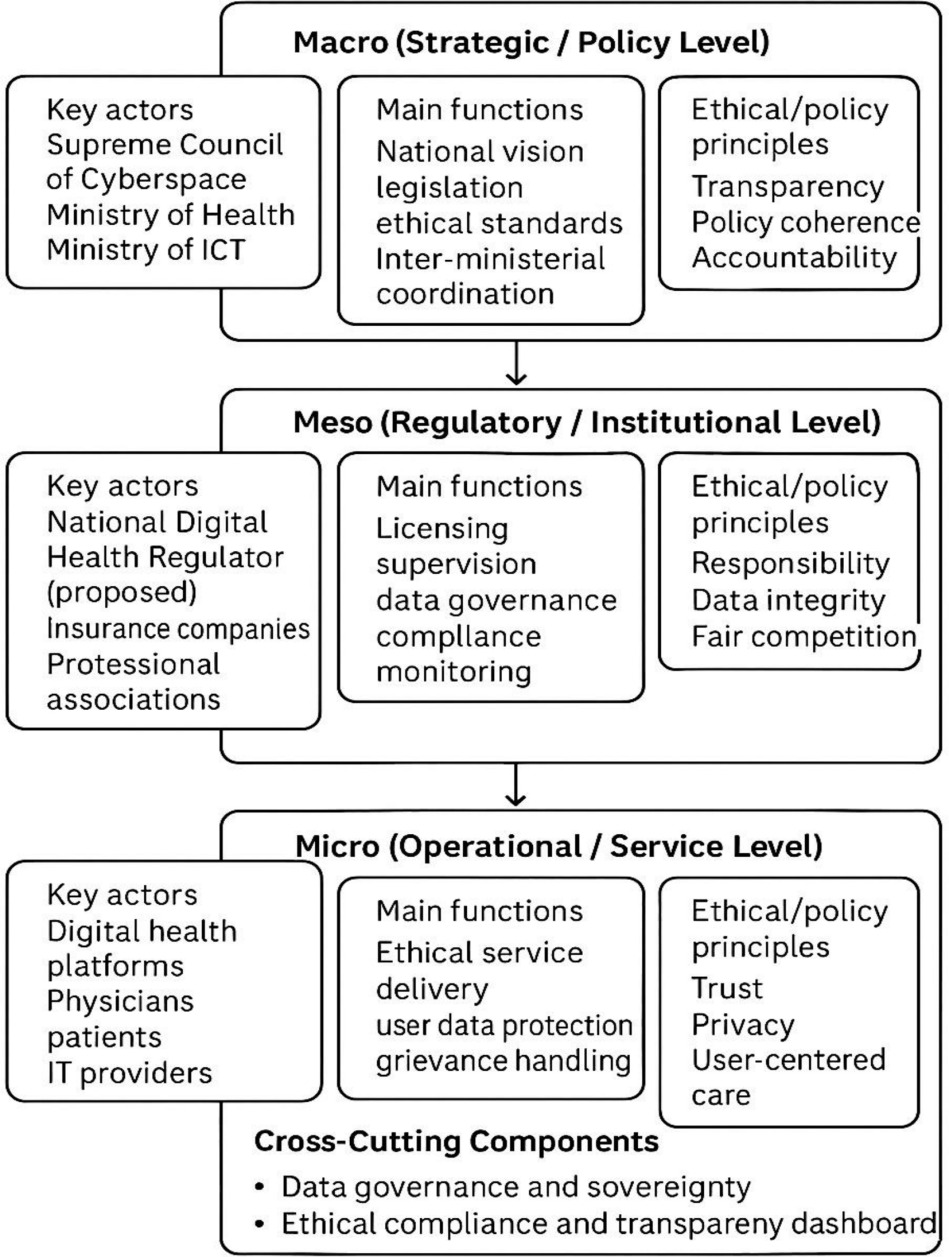
Integrated multi-level model of digital health governance in Iran. The figure presents an analytically derived, policy-oriented framework synthesising the identified governance gaps across three interconnected levels: macro (policy/strategic), meso (regulatory/institutional), and micro (operational/service). Five cross-cutting principles—transparency, responsibility, data integrity, stakeholder participation, and trust—link governance functions across levels. The model is informed by qualitative findings and is intended to support policy analysis and governance reform rather than to represent a directly validated empirical model

Building on the thematic findings, an integrated multi-level governance framework was analytically developed to synthesise the identified gaps (Fig. 2). The framework is informed by the empirical results and is intended as a policy-oriented model rather than a direct empirical finding.

At the macro level (strategic/policy), the framework emphasises national legislation, ethical standard-setting, and inter-ministerial coordination among institutions such as the Ministry of Health, the Ministry of ICT, and the Supreme Council of Cyberspace.

The meso level (regulatory/institutional) focuses on intermediary bodies, including regulatory authorities, insurance organisations, and professional associations, responsible for licensing, data governance, and oversight mechanisms.

The micro level (operational/service) encompasses digital health platforms, healthcare providers, and users, where governance principles are enacted through daily practices such as data protection, quality assurance, and complaint handling.

Across all levels, five cross-cutting principles—transparency, responsibility, data integrity, stakeholder participation, and trust—link governance functions and ethical practice. The framework provides a structured basis for addressing the governance gaps identified in this study.

## Discussion: Interpreting the governance gaps and implications for digital health reform

This study identified six interrelated governance gaps shaping the ethical and institutional performance of digital health services in Iran. Building on these empirical findings, the integrated framework (Fig. 2) offers an analytically derived, policy-oriented structure for understanding how regulatory, ethical, institutional, and economic factors interact across multiple governance levels. Rather than presenting the framework as a directly validated empirical model, it should be interpreted as a synthesis tool that translates qualitative insights into a coherent governance logic applicable to Iran and similar settings.

### Regulatory fragmentation and oversight gap

The findings highlight regulatory fragmentation as a foundational governance challenge, characterised by overlapping mandates among multiple ministries and councils and the absence of a unified licensing and monitoring mechanism. Similar patterns have been observed in other middle-income contexts where digital health innovation has advanced more rapidly than regulatory coordination [7, 9]. In such environments, fragmented oversight often leads to inconsistent enforcement and uncertainty for both providers and users. From a policy perspective, international experience suggests that greater coordination—whether through a centralised authority or harmonised inter-agency mechanisms—can improve transparency and accountability. However, the specific institutional form such coordination should take in Iran requires careful consideration of existing governance structures and political feasibility.

### Ethical and trust gap

The absence of clearly articulated and enforceable ethical standards for online medical services emerged as a major contributor to weakened professional accountability and declining user trust. Consistent with findings from studies in other regions [8, 10, 18], leaving ethical decision-making solely to individual platforms or practitioners risks inconsistency and opacity. Embedding digital health ethics within formal governance arrangements—such as professional guidelines, oversight mechanisms, and complaint-handling procedures—appears critical for strengthening legitimacy. In the Iranian context, the findings suggest that ethical governance is currently perceived as fragmented and discretionary, underscoring the need for more explicit institutional anchoring of ethical norms.

### Data governance gap

Data governance challenges—including unclear ownership, limited interoperability, and concerns about privacy protection—were reported across stakeholder groups. These issues mirror global debates on health data stewardship, particularly in settings where regulatory frameworks have not kept pace with platform-based data generation.

This aligns with global evidence showing that weak data governance frameworks and lack of interoperability are common barriers to effective digital health integration [19]. As van Panhuis et al. demonstrated, data-sharing barriers often stem from unclear ownership and regulatory fragmentation, limiting the reuse of health data and undermining privacy protections [19].

International guidance, such as WHO and OECD frameworks, emphasises that effective digital health systems depend on clear rules governing data access, storage, and accountability. The findings from this study suggest that, in Iran, ambiguities in data governance not only raise ethical concerns but also undermine user confidence and system sustainability.

### Quality and standardization gap

The lack of nationally recognised quality benchmarks and accreditation mechanisms for digital health services was found to undermine both patient safety and service credibility. Similar challenges have been documented in health services research, where inconsistent standards contribute to variable care quality and weak accountability [9]. In the absence of formalised quality indicators, both users and providers rely on subjective judgments, which may exacerbate trust deficits. The findings indicate that quality governance remains underdeveloped and insufficiently integrated into existing licensing and oversight arrangements.

### Institutional capacity gap

Limited institutional capacity—manifested in shortages of specialised expertise, weak inter-organisational coordination, and resistance to change—emerged as a cross-cutting constraint on governance effectiveness. Comparative research on digital transformation highlights that governance capacity depends not only on formal rules but also on institutional learning, leadership, and human resources [20, 21]. The Iranian case illustrates how gaps in digital ethics, health informatics, and data protection expertise can impede the translation of policy intentions into operational practice.

### Economic and sustainability gap

Economic fragility was identified as a key factor shaping ethical behaviour and long-term viability in the digital health sector. Uncertain pricing mechanisms, limited insurance reimbursement, and weak investment incentives place pressure on startups, sometimes discouraging investment in ethical safeguards. Evidence from low- and middle-income countries suggests that governance frameworks that align economic incentives with compliance and innovation are important for sustaining responsible digital health ecosystems [4, 7]. The findings indicate that ethical governance cannot be fully separated from broader economic and financing arrangements.

### Policy and practice implications

Taken together, the governance gaps identified in this study point to the need for more coherent and coordinated approaches to digital health governance. The proposed multi-level framework provides a conceptual roadmap for aligning ethical principles with governance functions across policy (macro), institutional (meso), and operational (micro) levels. At the macro level, clearer policy coordination and legal coherence are essential for reducing fragmentation. At the meso level, regulatory and professional bodies play a key role in operationalising ethical norms through licensing, oversight, and data governance mechanisms. At the micro level, platforms and service providers are central to implementing ethical practices that directly shape user experience and trust. While the framework does not prescribe a single reform pathway, it highlights leverage points where governance interventions may be most effective.

### Limitations and future research

This study has several limitations. First, the qualitative design and purposive sampling strategy limit the transferability of findings beyond the studied stakeholder groups. Perspectives of patients in remote areas, frontline clinicians, insurers, and some regulatory actors may be underrepresented. Second, although triangulation with user-generated comments strengthened analytic credibility, such data may be subject to selection and negativity bias, as users who post reviews are often those with particularly positive or negative experiences. Third, the analysis is grounded in Iran’s national context, and cross-country comparison was beyond the scope of this study.

Future research could employ mixed-method or comparative designs to examine how governance gaps manifest across different regulatory environments and stages of digital health maturity. Longitudinal studies may also help capture how ethical and governance frameworks evolve over time, particularly in response to emerging technologies such as AI-enabled health tools and large-scale data analytics.

## Conclusions

This study provides empirical and conceptual insights into the governance of digital health in Iran, identifying six interrelated governance gaps—regulatory and oversight, ethical and trust, data governance, quality and standardization, institutional capacity, and economic and sustainability. Together, these findings highlight how fragmented oversight, weak ethical accountability, and inadequate data stewardship constrain the legitimacy, efficiency, and sustainability of digital health transformation.

By synthesizing these gaps into an integrated multi-level governance framework, the study advances both theory and practice. Empirically, it demonstrates how governance weaknesses manifest in a rapidly evolving health technology environment. Theoretically, it proposes a model linking macro (policy), meso (regulatory), and micro (operational) levels through five ethical principles—transparency, responsibility, data integrity, stakeholder participation, and trust. Practically, it offers a policy-oriented roadmap to strengthen accountability, coherence, and ethical integrity in national digital health systems.

The framework has broader relevance for other low- and middle-income countries where innovation often outpaces institutional capacity. Embedding ethical governance across all levels of digital health policy and practice is critical to ensure that technological progress translates into equitable and trustworthy healthcare.

### Policy recommendations

Based on the findings and analytical framework, the following policy-relevant considerations are suggested.

**Establish a National Digital Health Authority** to unify oversight, streamline licensing, and coordinate inter-ministerial regulation.

**Develop a National Ethics Charter for Digital Health**, covering user consent, privacy, professional accountability, and conflict-of-interest management.

**Enact a Health Data Governance Act** defining ownership, localization, access rights, and protection standards in alignment with WHO and OECD guidance.

**Create national telehealth quality and accreditation standards** to ensure ethical and professional consistency in digital service delivery.

**Invest in institutional and human capacity building**, focusing on digital ethics, data stewardship, and governance leadership.

**Implement financial and policy incentives** to encourage ethical innovation, reimbursement mechanisms, and long-term sustainability.

### Final reflection

Governance and ethics form the backbone of sustainable digital transformation. Without coherent regulation, transparent data practices, and public trust, technological innovation risks amplifying inequities rather than resolving them. Strengthening digital health governance is therefore not merely an administrative reform—it is a moral and strategic imperative for building trustworthy, inclusive, and resilient health systems in Iran and beyond.

## Supplementary Information

Below is the link to the electronic supplementary material.

Supplementary Material 1

## Data Availability

The datasets generated and analyzed during the current study are not publicly available due to confidentiality agreements with participants but are available from the corresponding author on reasonable request.

## Abbreviations

BMC: BioMed Central
COREQ: Consolidated Criteria for Reporting Qualitative Research
eHealth: Electronic Health
ICT: Information and Communication Technology
LMICs: Low- and Middle-Income Countries
OECD: Organisation for Economic Co-operation and Development
WHO: World Health Organization
