# Supplementary material for: Bridging ethical and governance gaps in digital health: insights from users and startups in Iran
Source: BMC Health Serv Res. 2026 Apr 25;26:821. doi: 10.1186/s12913-026-14439-9 (PMC13270720; doi:10.1186/s12913-026-14439-9)
Supplement: Supplementary file 1 — Supplementary Material 1 [file 12913_2026_14439_MOESM1_ESM.docx]

**Supplementary File 1 – Interview Guide (Original Protocol)**

**Interview Guide Used in the National Project:**

Developing an Ethical Framework for Digital Health Businesses, (National Center for Cyberspace, Iran)

This semi-structured interview guide was originally developed and used in a national policy project commissioned by the **National Center for Cyberspace of Iran**.

The guide focuses on identifying ethical, legal, regulatory, and operational challenges experienced by digital health businesses.

**Section A: User-related Challenges**

1. At the beginning of your activity, what challenges did you face?
2. What are the most common problems you experience with users?
3. How are user complaints handled? Which unit in your organization reviews them?
4. In case of disputes between users and the platform, which authority do you refer to?
5. When financial or medical harm occurs, is it covered by insurance? What regulations exist for compensating damages? Who is responsible in telemedicine cases?
6. If an employee violates rules, are there specific sanctions?

**Section B: Oversight and Regulatory Processes**

1. Which licenses have you obtained and from which organizations?
2. Which institution supervises your company’s activities?
3. Who is responsible for monitoring app/website security, provider qualifications, advertising, pricing, etc.? How can users identify a trustworthy health platform?
4. Which authority determines service pricing? Is there a clear pricing guideline?
5. How is the security of financial, medical, and personal data ensured? Which protocols and approvals are in place?
6. How are service providers (e.g., physicians) verified?
7. Are the requirements of the national e-commerce trust mark (“Enamad”) sufficient for digital health businesses?
8. How can users directly contact responsible personnel?

**Section C: Privacy and Informed Consent**

1. Do you have a privacy policy? How was it developed?
2. Are user data used for non-medical purposes (e.g., advertising)? Is consent obtained?
3. How is informed consent collected (once at registration or during different steps)?
4. Can users withdraw consent?
5. When users uninstall the app or withdraw consent, are their data fully deleted?
6. Do you inform users about changes in data use purposes? How?
7. Are service logs (e.g., chats, calls, and video sessions) recorded? For how long?
8. Is there a way for providers to track whether the patient follows medical advice?

**Section D: Training**

1. Are physicians and staff trained in data security and proper use of the platform?
2. Are users provided with instructions on privacy protection or proper use?

**Section E: Special Populations**

1. What procedures exist for users who may lack decision-making capacity?
2. Can children use the service? Is parental supervision required? What regulations govern this?

**Section F: Ethical Guidelines**

1. Does your company have an ethical guideline?
2. What is your opinion about the draft national Ethical Charter for digital health businesses?
3. What challenges do you face in implementing ethical commitments?

**Section G: Open Questions**

1. Please describe any additional challenges or barriers your company faces
